# How many faces do people know?

**DOI:** 10.1098/rspb.2018.1319

**Published:** 2018-10-10

**Authors:** R. Jenkins, A. J. Dowsett, A. M. Burton

**Affiliations:** 1 Department of Psychology, University of York, York YO10 5DD, UK; 2 School of Psychology, University of Aberdeen, Aberdeen, UK

**Keywords:** face recognition, memory, social group size, mental representation

## Abstract

Over our species history, humans have typically lived in small groups of under a hundred individuals. However, our face recognition abilities appear to equip us to recognize very many individuals, perhaps thousands. Modern society provides access to huge numbers of faces, but no one has established how many faces people actually know. Here, we describe a method for estimating this number. By combining separate measures of recall and recognition, we show that people know about 5000 faces on average and that individual differences are large. Our findings offer a possible explanation for large variation in identification performance. They also provide constraints on understanding the qualitative differences between perception of familiar and unfamiliar faces—a distinction that underlies all current theories of face recognition.

## 1. Introduction

For most of human history, social groups have been small and widely dispersed [1,2]. This pattern has changed radically in recent centuries [3]. The rapid increase in population density implies changing demands on our ability to identify people we know (familiar faces) and people we do not know (unfamiliar faces). Cognitive research on face perception has revealed important differences in processing for familiar and unfamiliar faces [4,5]. Yet no one has established *how many* faces people know. In some ways, this is a puzzling omission. Numerosity is fundamental to quantitative research, and often propels theoretical and applied advances. For example, language research [6] and education policy [7] routinely cite vocabulary size—the number of words people know. Here, we report, to our knowledge, the first estimate of ‘vocabulary size' for facial identities.

As with abundance estimates in other domains (e.g. number of species on Earth [8], number of habitable planets [9]), there is a limit to the precision that can be achieved. Our aim is to converge on an order of magnitude. Anthropological research indicates that people maintain social networks of around 100–250 individuals [1], while forensic analyses often model trillions of unique face patterns [10]. As this range spans 10 orders of magnitude, a narrower estimate would usefully constrain theoretical development in face recognition.

For this study, we are not concerned with the number of faces people *could* know. That is a question of memory capacity [11]. Instead, we focus on the number of faces that people *actually* know. To address this question, it is essential to distinguish between faces and specific images of faces. If we encounter a particular person, and later see the same person again, the face image will be different [12]. For faces that we know, such incidental image changes are no barrier to identification. Indeed, image invariance is a hallmark of familiar face processing [13]. By contrast, identification of previously unseen faces can easily be disrupted by a change in image [14]. These observations demonstrate that knowing a face does not reduce to knowing a specific image [15,16].

It is also important to distinguish between knowing a person's face and knowing a person's name. These two forms of person knowledge readily dissociate [17,18]. For example, one might recognize the faces of fellow commuters, but never discover their names. Conversely, one might learn the names of famous authors, but never see their faces. In this study, we provided criteria specifying what counts as knowing a face. For recall, we stipulated that the participant should (i) be able to form a clear mental image of the face, or (ii) believe that they would recognize the face if they saw it. For recognition, participants should be able to recognize two different images of a known person.

## 2. Methodological approach and overview

**Figure 1.**
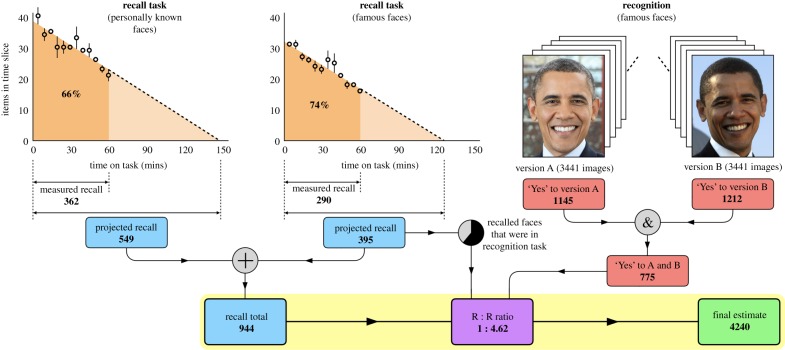
Combining different memory measures for personally known and famous faces. Recall estimates for each category are summed to produce a recall total (blue). Recall and recognition data for famous faces (red) are then compared to calculate a recall-to-recognition (R : R) ratio (purple). Increasing the recall total in this ratio leads to a final estimate (green). Error bars show residuals from the linear.

As it is not possible directly to assess the number of faces that people know, we decomposed the problem into several subcomponents (figure 1).

We begin by distinguishing two categories of known faces. We define *personally known* faces as those that the viewer acquired through direct social exposure. Friends, family and colleagues are examples of personally known faces. We define *famous faces* as the complement: faces that the viewer knows, but did not acquire through social exposure. Politicians, actors and musicians are typical examples of famous faces. To seed data collection, we measured cued recall for (i) personally known faces and (ii) famous faces in separate 1 h sessions (see §4). For both tasks, the rate at which new items were generated declined over the hour, but did not reach zero (figure 1). To estimate the point at which no new items would be generated, we extrapolated the trend lines to zero. For each task, projected recall refers to the estimated number of faces that participants would have recalled, given unlimited time.

Memory retrieval is highly fallible, even when supported with retrieval cues [19]. Given this fallibility, there are probably many faces that participants know, but which did not occur to them during testing. To estimate the proportion of known faces that were not recalled, we combined recall data with recognition data from the same participants. In this task, we showed participants the faces of 3441 public figures, and asked them whether or not they recognized each face (§4). By presenting two photos of each face (6882 images in total), we established which faces were recognized consistently in different images. Combining recall and recognition measures allowed us to calculate a *recall-to-recognition ratio*: for every one famous face recalled, how many were actually recognized? To adjust our recall measures in this proportion, we pooled the projected recall estimates for personally known faces and famous faces, and applied the recall-to-recognition ratio to the total. Based on this procedure, we estimate that people know around 5000. The range 1000–10 000 encompassed everyone we tested.

## 3. Discussion

Our estimate combines a number of assumptions. The first is that recall rates for personally known faces and famous faces continue to decline consistently beyond the 1 h testing period. Although previous studies of unconstrained recall generally involve shorter test sessions (less than 30 min), the reported trajectories are consistent with ours [20,21]. A steeper decline after 60 min would imply a lower final estimate, but note that our recognition data impose a hard lower bound on the famous face component. An alternative is that the decline in recall rate could stabilize into a long tail. Given that a long tail (by definition) adds new items slowly, it seems unlikely that it could amount to an order of magnitude difference. Importantly, our projected recall estimates from the laboratory task converge well with measured recall over a five-week period (§4c). This convergence provides additional support for our projections. A second assumption is that a recall-to-recognition ratio derived from famous faces also holds for personally known faces. It is conceivable that the ratio is different for these two categories of familiar faces. However, behavioural and neuropsychological studies suggest that similar memory mechanisms apply to both types. For example, both are characterized by image invariant recognition [22], and both are impaired in developmental [23,24] and acquired [25,26] prosopagnosia. Indeed, in our own data, recall measures for personally known faces and famous faces were positively correlated. Finally, one might argue that our familiarity criterion was too stringent. We required participants to recognize *both* images of a face in order to count the face as known. An alternative criterion would be to recognize *either* image. This less stringent criterion yields a mean of around 6000 and a range of around 1500–15 000, but does not capture the image invariance that characterizes familiar face recognition [5,13]. No fine-tuning of these assumptions delivers an estimate with an order of magnitude different from our estimate of 5000.

Our findings raise interesting questions about individual differences in vocabulary size for facial identities. We found substantial variation in every subtask, which echoes the variability seen in face identification performance [12,27,28]. These individual differences could reflect heterogeneity in visual cognition. For example, some individuals might attend to faces more than others, or encode them especially well. However, aspects of the social environment may also play a role. In particular, local population density could affect the frequency of face-to-face encounters [29], and the rate at which faces are acquired across the lifespan [30]. The current estimate provides a baseline against which to assess demographic and developmental moderators. It also provides a useful comparison with automatic face recognition systems, which may be trained on millions of identities [31–33].

We anticipate that the general method described here can be adapted to investigate individual differences in face recognition performance (by combining it with other psychometric assessments), and to trace the developmental trajectory of vocabulary size for facial identities (by testing different demographic groups). Future work on this topic may gain additional insights from Internet search and social network data. Finally, we note that there is nothing in our approach that confines it to the topic of face perception. Although we focus on faces in the current study, the same method could be used to quantify mental representations in other domains of cognition, such as familiar places [34] and objects [16].

## 4. Methods and results

### (a) Participants

Participants were 25 undergraduate or postgraduate students at the University of Glasgow and the University of Aberdeen (15 female, 10 male; mean age 24, age range 18–61 years). Based on pilot work, we anticipated a total of 5–6 h testing for each participant. Given the high time commitment, we recruited via email experienced experimental participants who had been reliable in previous studies. Participants received £30 basic payment plus performance-related pay for recall of famous faces, as described below.

### (b) Recall of personally known faces

Our goal in this task was to elicit as many items as possible from each participant. To be clear about task demands, and to foster consistency across participants, we provided written criteria specifying what counts as knowing a face. We stipulated that the participant should (i) be able to form a clear mental image of the face, or (ii) believe that they would recognize the face if they saw it. Of course, we had no means of verifying mental imagery, and participants could be mistaken about their own recognition abilities at this stage. Nevertheless, we found these criteria useful in guiding participants' understanding of the task. Importantly, we did not require participants to know the person's name. Naming is clearly separable from visual recognition [17,18], and we accepted uniquely identifying semantic descriptions (e.g. school janitor) in cases where the name could not be retrieved or was never known.

To structure participants' recall, and to assist them in conducting an exhaustive memory sweep, we provided response sheets (Microsoft Excel) that were organized into 14 headed columns: family, friends of family, own friends, family of own friends, school (including staff), colleagues, locals (neighbours etc.), retail staff, sports friends, social circles (e.g. church, pub), commuters, students, professionals (e.g. doctors, dentists), and people met on a holiday or trip. We also encouraged participants to divide their lives into autobiographical chapters (perhaps based on where they were living or working) and to use a separate worksheet for each such chapter. The intention here was to maximize recall by prompting participants to consider all the different social settings in which they might have acquired personally known faces, and to repeat this systematically for each autobiographical chapter.

Participants completed this task individually and in silence, entering items into the spreadsheet continuously for 60 min. We automatically saved the spreadsheet data every 5 min, allowing us to reconstruct the rate at which new items were generated. The average number of personally known faces recalled in this way was 362 (s.d. = 93; range = 167–524). We limited recall sessions to 60 min to spare participants fatigue. However, this arbitrary limit raises the question of whether participants had exhausted their recall, or would they have continued to generate new items, given more time. To address this question, we analysed the rate at which participants recalled new faces during the 1 h session. Participants generated an average of 40 items in the first 5 min time-slice, slowing to 21 items in the final time-slice. Two aspects of the data are immediately apparent. First, an hour was not enough to exhaust recall fully, as participants were still generating new items at the end of the hour. Second, recall rate declined approximately linearly over the hour (figure 1). This decline allowed us to estimate the time required to exhaust recall by projecting the trend line to the zero crossing (145 min). Since the area of region 0–60 min is approximately 66% the area of region 0–145 min, we estimate that the number of personally known faces recalled in the hour (measured total) is 66% of the total they would reach given unlimited time (projected total M = 549; s.d. = 141; range = 253–794).

### (c) Comparison with pilot data

In pilot testing, we recruited 10 volunteers to complete a pen and paper version of the same recall task over a five-week period outside of the laboratory. The projected recall estimate from the laboratory task converged well with the measured recall estimate in this pilot test (M = 429; s.d. = 141; range 230–718).

### (d) Recall of famous faces

The procedure for recall of famous faces was the same as for personally known faces, except for the following changes. First, we changed the recall cues so that they were appropriate for famous faces. Twelve new categories—arts and media, business, fashion, film, historical figures, music, politics, royalty, science, sports, TV and other—were now presented on separate worksheets. Within each worksheet, we included headed columns for relevant subcategories (e.g. drama, comedy, cookery, etc., within the TV category). We also provided episodic prompts such as, ‘What films have you seen recently?'. Once again, the aim was to maximize recall by prompting participants to consider all the settings in which they might have been exposed to famous faces.

Our second change was to add performance-related pay. One concern with long test sessions (60 min) is a loss of motivation in the participant. Motivational factors may be important in interpreting the rate at which participants generate new items. A slowdown could mean that the participant is running out of items, consistent with an exhaustive memory search. Alternatively, it could mean that the participant is losing motivation. To counteract the loss of motivation, we introduced a cash incentive in addition to standard participant payments (we did not apply this pay scale to recall of personally known faces because we did not want to incentivize generation of spurious items). The cash incentive began at 1p per item, and rose by 1p per item for every 100 items recalled. Thus, the reward for the next 100 items was always higher than the reward for the preceding 100 items.

The average number of famous faces recalled in this way was 290 (s.d. = 69; range = 169–407). We note that participants recalled significantly more personally known faces (M = 362) than famous faces (M = 290) (*t*_24_ = 3.89, *p* < 0.001). This trend was present for 21 of our 25 participants. To test for dependence between the recall measures for personally known faces and famous faces, we calculated Pearson's correlation coefficient from participants' recall scores (*r*_23_ = 0.38, *p* < 0.05). The positive correlation between these measures indicates that participants who recalled many personally known faces also recalled many famous faces, and vice versa. Participants were financially motivated to recall famous faces because they received per-item payment. Given that recall rate was actually higher for personally known faces, and performance in the two tasks was positively correlated, we found no evidence that participants were less motivated when recalling personally known faces.

As with the previous task, we analysed the rate at which participants recalled new faces during the 1 h session. Participants generated an average of 31 items in the first 5 min time-slice, falling to 16 items in the final time-slice. Once again, the data follow an approximately linear decline. Extrapolating this trend line to the zero crossing (120 min) implies that the number of famous faces recalled in the hour (measured total = 290) is 74% of the total they would reach given unlimited time (projected total M = 395; s.d. = 94; range = 230–553).

### (e) Famous face database

Given the fallibility of recall [19], there are probably many faces that our participants could recognize perfectly well, but which did not occur to them during the recall sessions. For this reason, we next conducted a recognition test for famous faces, which could be used to make a final estimate of people's vocabulary size of faces. To obtain a suitably large set of test items, we pooled the names of public figures from 12 members of our laboratory group (undergraduate students, postgraduate students and faculty members), none of whom participated in the main study (see Acknowledgements). Each of these contributors was asked to think of public figures whose faces they knew, and to enter the names of these public figures into a shared online spreadsheet. We intended this exercise to be as exhaustive as possible, and we expected it to be time-consuming. Instead of completing it in one sitting, contributors were asked to integrate it into their normal workflow as a background task. After three months, we closed contributions because none of the contributors had added any new items for two consecutive weeks.

The resulting list contained the names of 3441 public figures. We do not claim that this is in any sense a complete list of famous people. All that is required is that the list is sufficiently large to include many items that were recalled by participants, and to capture fine-scale differences in performance. Our next step was to collect images of these public figures' faces. We collected two different photographs of each public figure by entering their names as search terms in Google Image. To systemize sampling, and to ensure recognizable likenesses [12], we selected the first two colour photos of the corresponding person that: (i) exceeded 200 pixels in height, (ii) showed the face in roughly frontal aspect, and (iii) were free from occlusions (6882 images in total). All photos were cropped to show the head region only and resized to 400 pixels high × 320 pixels wide for presentation.

### (f) Recognition performance

We used these ambient face images to construct two versions of a face recognition test. Version A contained the first image of each person (3441 images); version B contained the second image of each person (also 3441 images). In each test, the 3441 images were presented sequentially on screen, with a different random order for each participant. For each image, the participants' task was to indicate via keypress whether or not they knew the depicted person (‘do you recognise this face? (Y or N)'). The task was self-paced with no time limit, and each image remained on screen until response. Each participant completed both version A and version B of the test in separate approximately 2 h sessions (approx. 4 h in total).

We intended ‘Yes' responses to signal genuine face recognition. However, any given ‘Yes' response could instead reflect: (i) recognition of the image only, not the person, (ii) a feeling of familiarity without recognition, (iii) response to task demands (if participants suspect that they are not making enough ‘Yes' responses), or (iv) motor error. Image recognition is not the subject of this study, and cases (ii)–(iv) represent different types of false alarm. Only in genuine person recognition would we expect the participant to demonstrate image invariance, by responding ‘Yes' to both images of that person (version A and version B). For this reason, our analysis focuses on those cases that demonstrate image invariance.

Performance in the two versions of the task was very similar. In both versions of the task, participants recognized about 30% of the items on average (1145 in version A; 1211 in version B). As our main interest was face recognition, as distinct from image recognition, we carried forward only those faces that were recognized in version A and version B (775). This metric confirms that levels of recognition were much higher than levels of recall. Face recognition without recall was common, whereas face recall without recognition was rare or absent [17]. However, the recognition set was not a simple super-set of the recall set: 61% of items recalled also appeared in the recognition set, while 39% did not. There were again large individual differences in the number of faces recognized in each version of the task (approx. 400–2000). These differences were stable in that performance was highly correlated across the two versions (*r* = 0.96). Participants who recognized many items in version A also recognized many items in version B, and vice versa.

### (g) Combining recall and recognition estimates

To estimate the total number of faces known, we combined recall and recognition data in the following way. First, for each participant, we compared (i) those famous faces in the recognition database which they had recalled, and (ii) those famous faces in the recognition database which they had recognized. This comparison results in a recall-to-recognition ratio of 1 : 4.62. We then summed the recall estimates for personally known faces (projected recall M = 549) and famous faces (whether they were in the recognition database or not; projected total M = 395) to arrive at an estimated total for recall (M = 944; s.d. = 197; range = 583–1286). Finally, we increased this estimate in the recall-to-recognition ratio (1 : 4.62) to reach a final estimate of 4240 known faces (s.d. = 2136; range = 1031–8579). This exact number implies a level of precision that we do not have. Our proposal is to round it up to 5000, and to note that all of the participants we tested fell within the range 1000–10 000 (see the electronic supplementary material).

## Supplementary Material

Participant data

## Data accessibility

Data available from the Dryad Digital Repository: https://doi.org/10.5061/dryad.7f25j43 [35].
