# Supplementary material for: How many faces do people know?
Source: Proc Biol Sci. 2018 Oct 10;285(1888):20181319. doi: 10.1098/rspb.2018.1319 (PMC6191703; doi:10.1098/rspb.2018.1319)
Supplement: Participant data [file rspb20181319supp1.pdf]

Demographics			Measured Recall		Projected Recall		Recognition			Recall-to-recognition Ratio		Apply r:r Ratio	
Participant	Gender	Age	Personal	Famous	Personal	Famous	Version A	Version B	A & B	Recalled in DB	r:r Ratio	Recall total	Rt x r:r Ratio
1	F	22	524	327	794	444	1470	1492	990	206	4.81	1239	5952
2	F	61	225	178	341	242	1935	2035	1018	122	8.34	583	4864
3	F	27	226	262	342	356	1040	934	610	162	3.77	699	2631
4	M	20	443	313	671	425	829	634	366	224	1.63	1097	1792
5	F	19	167	314	253	427	1133	1321	564	154	3.66	680	2490
6	M	22	478	244	724	332	1271	1670	882	144	6.13	1056	6468
7	F	19	436	264	661	359	812	945	572	165	3.47	1020	3534
8	M	18	437	170	662	231	1680	1741	859	101	8.50	893	7597
9	F	20	326	194	494	264	411	469	292	75	3.89	758	2950
10	M	22	278	244	421	332	401	473	333	159	2.09	753	1577
11	F	22	360	274	546	372	606	673	327	120	2.73	918	2501
12	M	21	393	361	596	491	1930	1789	1453	246	5.91	1086	6416
13	F	21	338	169	512	230	490	588	324	99	3.27	742	2428
14	F	47	424	372	643	506	509	536	329	226	1.46	1148	1671
15	F	20	273	285	414	387	1239	1315	1029	229	4.49	801	3600
16	M	21	389	407	589	553	1796	1736	1427	244	5.85	1143	6683
17	M	22	437	396	662	538	508	543	231	269	0.86	1200	1031
18	F	22	420	308	636	419	1292	1366	999	225	4.44	1055	4685
19	F	23	419	310	635	421	1156	1210	812	178	4.56	1056	4819
20	F	22	346	333	524	453	1300	1280	918	262	3.50	977	3423
21	F	21	495	394	750	536	1232	1618	943	203	4.65	1286	5972
22	F	23	309	296	468	402	599	717	434	175	2.48	871	2159
23	M	22	301	279	456	379	1803	1942	1335	130	10.27	835	8579
24	M	27	381	337	577	458	1189	1388	921	144	6.40	1035	6622
25	M	22	236	231	358	314	1996	1873	1404	170	8.26	672	5547
	**Mean**	**24.24**	**362**	**290**	**549**	**395**	**1145**	**1212**	**775**	**177**	**4.62**	**944**	**4240**
	SD	9.21	93	69	141	94	518	520	389	54	2.39	197	2136
	Min		167	169	253	230	401	469	231			583	1031
	Max		524	407	794	553	1996	2035	1453			1286	8579

[redacted]

Participant	Less stringent recognition criterion (either image A or image B)					
	No-No	Alt. Recog	Alt. Ratio	Alt. Pers	Alt. Fam	Alt. Total
1	1469	1972	9.57	5016	3130	8146
2	1018	2423	19.86	4469	3535	8004
3	2077	1364	8.42	1903	2206	4109
4	2344	1097	4.90	2170	1533	3702
5	1551	1890	12.27	2050	3854	5903
6	1382	2059	14.30	6835	3489	10324
7	2256	1185	7.18	3131	1896	5027
8	879	2562	25.37	11085	4312	15397
9	2853	588	7.84	2556	1521	4077
10	2900	541	3.40	946	830	1776
11	2488	953	7.94	2859	2176	5035
12	1175	2266	9.21	3620	3325	6945
13	2687	754	7.62	2574	1287	3861
14	2725	716	3.17	1343	1179	2522
15	1916	1525	6.66	1818	1898	3716
16	1335	2106	8.63	3358	3513	6870
17	2621	820	3.05	1332	1207	2539
18	1782	1659	7.37	3097	2271	5368
19	1887	1554	8.73	3658	2706	6364
20	1779	1662	6.34	2195	2112	4307
21	1534	1907	9.39	4650	3701	8351
22	2559	882	5.04	1557	1492	3049
23	1031	2410	18.54	5580	5172	10752
24	1785	1656	11.50	4382	3876	8257
25	976	2465	14.50	3422	3350	6772
**Mean**	**1880**	**1561**	**9.63**	**3424**	**2623**	**6047**
SD	642	642	5.40	2154	1152	3091
Min						1776
Max						15397
